# Clinical Impact and Prognostic Role of KRAS/BRAF/PIK3CA Mutations in Stage I Colorectal Cancer

**DOI:** 10.1155/2018/2959801

**Published:** 2018-06-19

**Authors:** Luca Reggiani Bonetti, Valeria Barresi, Antonino Maiorana, Samantha Manfredini, Cecilia Caprera, Stefania Bettelli

**Affiliations:** ^1^Department of Diagnostic Medicine and Public Health, Section of Pathology, University of Modena and Reggio Emilia, Modena, Italy; ^2^Department of Pathology, University of Messina, Messina, Italy

## Abstract

Stage I colorectal carcinoma has excellent prognosis, with 5-year survival rate up to 95%. The occurrence of lymphovascular invasion, tumor budding, high number of PDC, or lymph node micrometastases is associated with tumor progression. The aim of this study was to evaluate the mutational status of 62 stage I colorectal carcinomas (CRC) (taken from 37 patients surviving more than five years since the initial diagnosis and from 25 patients who died of disease) and to correlate it with histopathological features and the clinical outcome. Mutations of *KRAS*, *NRAS*, *BRAF*, and *PIK3CA* genes were analyzed through Myriapod Colon Status Kit, using the high-throughput genotyping platform Sequenom MassARRAY System. Mutations in those genes were found in 31 cases (50%) and mainly in those with poor prognosis. The most frequent mutations occurred at codons 12 and 13 of the *KRAS* gene (40% of cases). We found concomitant *PIK3CA* mutations in 5 cases (8%). The presence of *PIK3CA* mutations was mainly observed in tumors with poor prognosis and with unfavorable histopathological prognostic features. High PDC grade (*P* = 0.0112), the presence of tumor budding (*P* = 0.0334), LVI (*P* < 0.0001), *KRAS* mutations (*P* = 0.0228), *PIK3CA* mutations (*P* = 0.0214), multiple genetic mutations in *KRAS* and *PIK3CA* genes (*P* = 0.039), and nodal micrometastases (*P* < 0.0001) were significant prognostic variables for CSS. The presence of LVI was the only independent and statistically significant prognostic variable for CSS in our cohort of pTNM stage I CRCs. The analysis of *KRAS/PIK3CA* mutational status may be used to identify patients with stage I CRC at high risk of bad outcome and who may need additional treatments, including biological therapies.

## 1. Introduction

It is well known that pTNM stage I (pT1N0M0 and pT2N0M0) colorectal carcinoma (CRCs) has an excellent prognosis, with 5-year survival rate up to 95% [1]. Therefore, patients with pTNM stage I CRC are not submitted to any adjuvant treatment after surgery [1]. However, in a small percentage of cases, they develop recurrences or metastases during the follow-up, and this event seems to be more frequent in cases showing lymphovascular invasion (LVI), tumor budding (TB), a high number of poorly differentiated clusters (PDC) in the primary tumor, or micrometastases in regional lymph nodes [2–5].

Up to now, only few studies have investigated the molecular profile of pTNM stage I CRC [6–8]. According to those studies, the percentage of mutated tumors ranges between 25% and 57%, and mutations mainly involve the KRAS gene in this subset of CRCs [6–8].

In this study, we aimed to investigate whether there is any correlation between the molecular profile and clinical outcome of pTNM stage I CRCs and whether mutations in *KRAS*, *BRAF*, *NRAS*, and *PIK3CA* genes are associated with histopathological features in this group of tumors.

## 2. Materials and Methods

### 2.1. Selection of Cases and Histological Examination

By a specialized Colorectal Cancer Registry instituted in Modena in 1984 [9], we identified all patients with stage I CRCs diagnosed between January 1984 and December 2004 (518 cases) and, among them, we selected those who died of disease (DOD) during the follow-up (37 cases). Paraffin blocks of the tumors and the relative haematoxylin and eosin- (H&E-) stained slides, stored in the archives of the Pathologic Anatomy of the University of Modena and Reggio Emilia, were available for only 25 of 32 patients (group A). This group of patients was matched with a group of 32 patients with stage I CRCs who were alive or who died of independent diseases (DOID) after a follow-up time longer than sixty months (group B). Cases in group B were consecutive stage I CRCs that fulfilled the inclusion criteria (at least 60-month follow-up) and with available paraffin blocks. All cases were anonymously collected.

Pathological features, including tumor size (maximum diameter in centimeters), tumor border configuration (expanding or infiltrating), WHO histological grade [10], pTNM stage [11], TB, LVI, grading based on the counting of PDC [12], and the presence of lymph node micrometastases (MM) [13], were available in all cases.

According to their location, the tumors were divided into 3 groups: (1) tumors located in the right colon, including the cecum and ascending and transverse colon; (2) tumors located in the left colon, including descending and sigmoid colon; and (3) tumors located in the rectum.

In accordance with the pathological staging at diagnosis (pTNM stage I) and the current oncological guidelines, none of the patients had been submitted to adjuvant chemoradiotherapy after surgical resection.

### 2.2. Molecular Analysis

DNA was extracted from representative 10 *μ*m-thick sections cut from formalin-fixed and paraffin-embedded blocks of each tumor sample containing at least 50% tumor cells. Extraction was performed with QIAamp DNA Mini Kit (Qiagen, Hilden, Germany), and DNA was quantified with Xpose-NGS (Trinean NV, Gentbrugge, Belgium). Mutations were detected in genome-amplified DNA using the high-throughput genotyping platform Sequenom MassARRAY System (Sequenom, San Diego, CA, USA) and the Myriapod Colon Status Kit (Diatech Pharmacogenetics, Italy) following the manufacturer's protocol. This molecular array allows to identify the most important mutations of *KRAS* (codons 12, 13, 59, 61, 117, and 146), *NRAS* (codons 12, 13, 18, 59, 61, 117, and 146), *BRAF* (codons 594, 600, and 601), and *PIK3CA* genes (codons 38, 81, 88, 93, 108, 118, 345, 420, 539, 542, 545, 546, 549, 1021, 1025, 1043, 1047, and 1049). In brief, 25 ng/*μ* DNA of each specific primitive tumor was amplified through multiplex PCR, and then unincorporated nucleotides were inactivated by shrimp alkaline phosphatase (SAP). A single-base extension reaction was performed using extension primers that hybridize immediately adjacent to the mutations and a custom mixture of nucleotides. Salts were removed by the addition of a cation exchange resin. Multiplexed reactions were spotted into SpectroCHIP II arrays, and DNA fragments were resolved by MALDI-TOF on the Compact Mass Spectrometer (Sequenom, San Diego, CA) with a limit of detection of 5%. Data were evaluated using MassARRAY Typer Analyser software 4.0, which allows to identify mutated alleles by comparing the ratio of the wild-type peak of all suspected mutants and to generate a specific report.

### 2.3. Statistical Analysis

Fisher exact and chi-squared tests were used to assess the statistical association between mutational status of the tumor and the clinicopathological parameters. Median age of the patients (68 years) and median size of the tumors (3 centimeters) were used as cutoff values for statistical analyses.

Cancer specific survival (CSS) was assessed by the Kaplan-Meier method, with the date of primary surgery as the entry date. CSS was characterized as the length of survival to death from CRC or to the last follow-up date.

The Mantel-Cox log-rank test was applied to assess the strength of association between CSS and each of the parameters (age and gender of the patient, size of the tumor, WHO histological grade, PDC grade, pT stage, tumor border configuration, TB, LVI, and MM) as a single variable.

Subsequently, a stepwise multivariate analysis (Cox regression model) was utilized to determine the independent effect of each variable on survival. Multivariate analysis was carried out by using stepwise method and including only clinicopathological variables with significant prognostic value at univariate analyses.

A probability (*P*) value less than 0.05 was considered statistically significant. Statistical analysis was done using MedCalc 12.1.4.0 statistical software (MedCalc Software, Mariakerke, Belgium).

### 2.4. Ethical Issues

All procedures were performed in accordance with the Helsinki Declaration. Ethical issues were discussed with the Local Ethics Committee. No formal approval was necessary to perform the histological review and molecular analyses.

## 3. Results

Clinical and pathological features of 62 tumors included in the study are summarized in Table 1. 31 tumors (50%) had mutations in *KRAS*, *BRAF*, and *PI3KCA* genes; details are listed in Table 2.

### 3.1. KRAS Mutations

**Figure 1:**
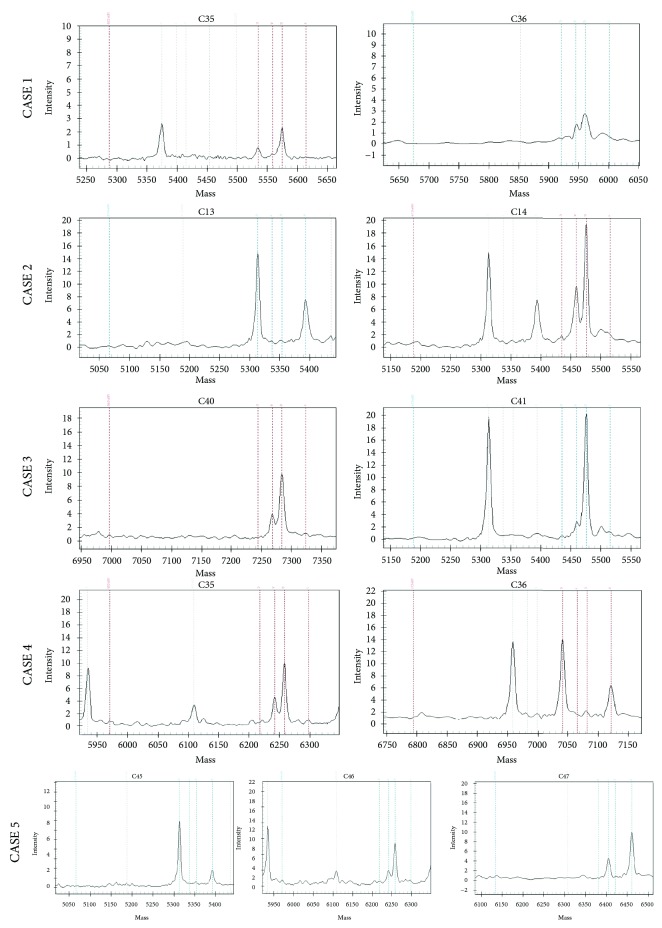
KRAS and PIK3CA MassArray (Sequenom) graphic assay. pTNM stage I CRCs with unfavourable clinical outcome showed frequently multiple KRAS mutations involving unusual codons and KRAS mutations associated to PIRKCA mutations.

28/62 cases (45%) had mutations in the *KRAS* gene. Among those, 22 (78%) had a single *KRAS* mutation (G13D in 7 cases, G12D in 6 cases, G12V in 5 cases, G12C in 1 case, G12F in 1 case, A146T in 1, and Q61H in 1), while 6 showed multiple mutations. In detail, 5 cases had also *PIK3CA* mutations (R108H in 2 cases, E545K in 2 cases, and R88K in 1), and 1 had two additional *KRAS* mutations (G12D + G13D + G117N). *KRAS* mutations were significantly more frequent in the group of DOD patients (*P* = 0.019) (Table 3) Figure 1.

### 3.2. PIK3CA Mutations

*PIK3CA* mutations were found in a total of five cases (Table 2), all of which showed *KRAS* mutations as well. Mutations were E545K in 2 CRCs, R108K in 2, and R88Q in 1 (Table 2). The presence of *PIK3CA* mutations was significantly more frequent in female patients (*P* = 0.01) and in cases with nodal micrometastases (*P* = 0.017) and PDC G3 (*P* = 0.001) (Table 3).

All but one patient with *PIK3CA*-mutated tumor died of CRC; the only one who was alive 168 months since the initial diagnosis had *PIK3CA* R88Q mutation.

### 3.3. NRAS Mutations

*NRAS* mutations were not identified in any of the cases.

### 3.4. BRAF Mutations

*BRAF* V600E mutation was found in 3 cases: 2 were from DOD patients, while one was from a DOID patient, who was alive 167 months since the initial diagnosis. Due to the limited number of cases with *BRAF* mutations, statistical association with other parameters was not investigated. However, all 3 mutated cases were localized in the right colon (Table 3).

### 3.5. CRC with Multiple Mutations

CRCs with multiple mutations (*KRAS* + *PIK3CA* or multiple *KRAS* mutations) occurred mainly in female patients (*P* = 0.004) and in DOD patients (*P* = 0.006) and had significant association with lymph node micrometastases (*P* = 0.011) and high PDC grade (*P* = 0.004). Multiple mutations were more frequent in CRCs showing LVI, though statistical significance was not reached (Table 3).

### 3.6. Correlations between CSS and Clinicopathological Parameters or Mutational Status

**Figure 2:**
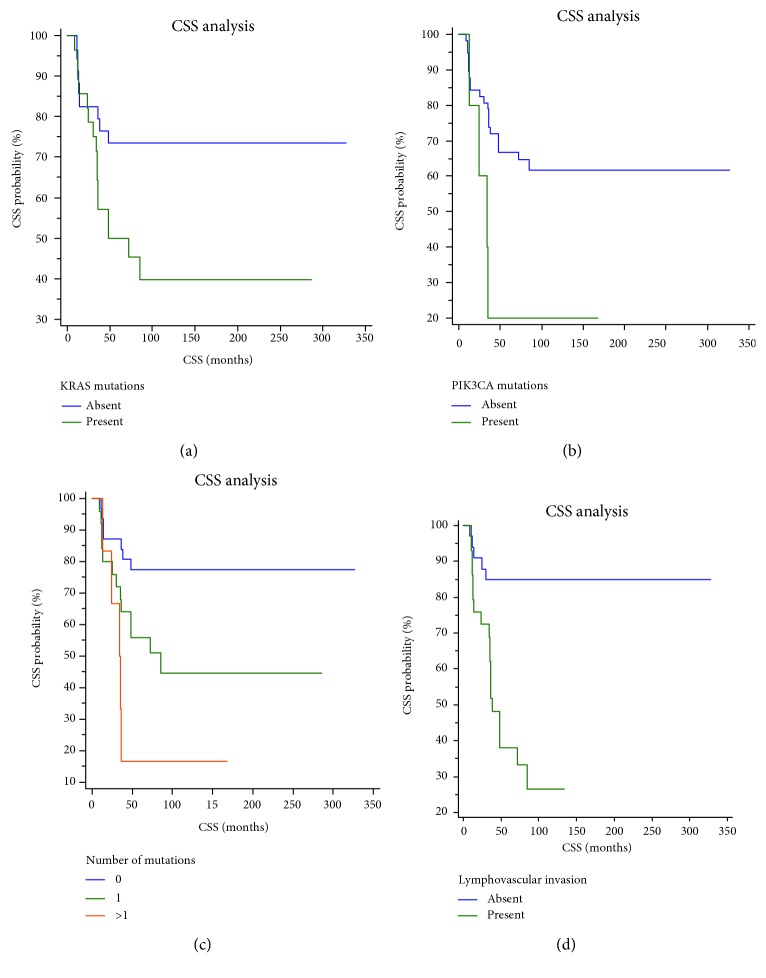
(a–c) Kaplan-Meier curves showing CSS of patients with pTNM stage I CRC according to mutational status of KRAS, BRAF, and PIK3CA genes. Patients having CRC with (a) KRAS, (b) PIK3CA, or (c) 2 or 3 mutations had significantly lower CSS, compared to patients with CRC with absent or 1 mutation. (d) Kaplan-Meier curves showing CSS of patients with pTNM stage I CRC according to the presence of LVI. Patients having CRC with LVI had significantly lower CSS compared to patients with CRC not having LVI.

Univariate analyses showed that high PDC grade (*P* = 0.0112), the presence of tumor budding (*P* = 0.0334), LVI (*P* < 0.0001), *KRAS* mutations (*P* = 0.0228), *PIK3CA* mutations (*P* = 0.0214), multiple genetic mutations in *KRAS* and *PIK3CA* genes (*P* = 0.039) (Figure 2), and nodal micrometastases (*P* < 0.0001) (Figure 2(b)) were significant prognostic variables for CSS (Table 4). The presence of LVI was the only independent and statistically significant prognostic variable for CSS in our cohort of pTNM stage I CRCs (Table 4) (Figure 2(d)).

## 4. Discussion

In this study, we investigated the molecular profile of a cohort of stage I CRCs and assessed its association with patient's prognosis and various clinicopathological variables. While the molecular profile of advanced CRC has been largely investigated to predict response to biological drugs [14–16], the mutational status of early CRC (stage I) has been rarely analyzed [6–8, 17, 18] and only in studies including stage I CRCs in heterogeneous cohorts of tumors at different pTNM stages [6–8, 17, 18]. Therefore, to the best of our knowledge, this represents the first study focused on the molecular profile of stage I CRC.

Our results can be summarized as follows: (1) mutations in the genes involved in RAS/MAPK and PI3K-PTEN-AKT signalling pathways were found in 50% of stage I CRC and mainly in cases with poor prognosis rather than in cases with favourable outcome (72% versus 40%), (2) the most frequent mutations observed in stage I CRC occurred at codons 12 and 13 of the *KRAS* gene (G13D, G12D, and G12V mutations), (3) *PI3KCA* mutations were strongly associated with *KRAS* mutations, and (4) synchronous *KRAS* and *PIK3CA* mutations or multiple *KRAS* mutations at codons 12 and 13 were mainly found in tumors with poor prognosis and with unfavorable histopathological prognostic features.

Hence, our findings confirm previous evidence that mutations at codons 12 and 13 (G13D, G12D, and G12V) of the *KRAS* gene are the most frequent mutations in stage I CRC [6–8, 17, 18], while *NRAS*, *BRAF*, and *PIK3CA* mutations are rare in this subgroup of CRC [6–8, 17].

The prognostic role of *KRAS* mutations in stage I CRC was previously investigated in two studies that showed that mutations in this gene are associated with shorter overall survival and recurrence-free survival [6, 18], while the specific prognostic role of *PIK3CA* mutations in stage I CRC had not been analyzed thus far. Our findings showed a significant association between *KRAS* or *PIK3CA* mutations and shorter CSS. Interestingly, the presence of multiple *KRAS* mutations and that of *KRAS/PIK3CA* bimutations were significant negative prognostic factors in our patients with stage I CRC. Li et al. [17] previously demonstrated that *KRAS/PIK3CA* bimutations are significantly more frequent in patients with stage IV CRC, compared to lower-stage carcinomas. *KRAS/PIK3CA* bimutations may induce metastasization through a synergic effect in the activation of PI3K-AKT pathway [17, 19, 20]. Since *KRAS* and *PIK3CA* mutations seem to be early events in colorectal carcinogenesis [21], we may hypothesize that early-stage CRCs with bimutations are more likely to behave as stage IV tumors and to develop distant metastases and worse outcome during the follow-up. Besides, in a previous study, we already showed the association between *KRAS/PIK3CA* bimutations and nodal metastases or lymphovascular invasion [22]. Further supporting the tendency of bimutated or trimutated stage I CRC to metastasization is that, in our cohort, this subgroup of tumors had significantly higher frequency of PDC G3 and nodal micrometastases. Indeed, several studies showed that PDC G3 CRCs have high metastatic potential at any pTNM stage [23]. In addition, the presence of nodal micrometastases in bi- or trimutated CRCs indicates disease diffusion in spite of pTNM stage I. Different from our previous study [24] on a cohort of CRCs at pTNM stages I, II, III, and IV, the presence of a single *KRAS* mutation was not associated with PDC grade or tumor budding in this group of pTNM stage I CRCs. Studies on a larger cohort are needed to clarify whether the association between *KRAS* mutations and PDC grade is stage-dependent or not.

Although further studies are necessary to corroborate these findings, patients with stage I CRC showing this biomolecular profile—duple or triple mutations—might be candidate to adjuvant treatments in the aim to prevent unfavorable outcome. The association between *KRAS*/*PIK3CA* multiple mutations and PDC G3 may be used as a screening tool to select cases with high probability to have *KRAS/PIK3CA* bimutations or *KRAS* multiple mutations and which may be referred to molecular analyses. Indeed, in order to avoid costs related to extensive molecular analyses on stage I CRC, evaluation of *KRAS* and *PIK3CA* mutational status may be limited only to cases showing PDC G3 at histological examination. Besides, the identification of *PIK3CA* mutations might also have therapeutic implications. Indeed, there is some evidence that CRC with *PIK3CA* mutations could be treated with PI3K/mTOR inhibitors [25].

Interestingly, the occurrence of LVI emerged as the only significant and independent prognostic variable in our patients with stage I CRC, as we already found in a previous study [26]. Interestingly, two of the pT1 CRCs included in our cohort who died of disease during the follow-up exhibited multiple synchronous *KRAS* mutations. Therefore, the assessment of *KRAS/PIK3CA* mutational status may be particularly important to predict prognosis of patients with pT1 CRC submitted to endoscopic conservative treatments in the aim to identify patients at high risk of metastatic diffusion and worse outcome, who could be submitted to surgery and adjuvant treatments.

In conclusion, this is the first study specifically analyzing the molecular profile of stage I CRC. The presence of *KRAS* mutations, that of simultaneous mutations in *PIK3CA* gene, or that of multiple *KRAS* mutations was significantly associated with shorter CSS. *PIK3CA* or multiple *KRAS* mutations were associated with nodal micrometastases and PDC G3 as well. If our findings are confirmed in further studies, the analysis of *KRAS/PIK3CA* mutational status may be used to identify patients with stage I or pT1 CRCs at high risk of worse outcome, who may need additional treatments.

## Figures and Tables

**Table 1:** Clinicopathological characteristics of the 62 CRCs at pTNM stage I.

Clinicopathological variables	Total patients (*n* = 62)	Alive/DOID patients (*n* = 37)		DOD patients (*n* = 25)
M/F	35/27	22/15		13/12
Mean age	69.5 years	68.7 years		70.84 years
Age range	48–90 years	48–85 years		55–90 years
Right colon	11	8		3
Left colon	15	11		4
Rectum	26	18		8
Mean size of the tumor (cms)	3.28	3.23		3.45
Size range of the tumor (cms)	1–6.5	1–5.5		1.5–6.5
pT1/pT2	4/58	1/36		3/22
Micrometastases (present/absent)	17/45	0/37		17/8
Tumor border configuration (expansive/infiltrative)	18/45	14/23		4/21
WHO grading (G1/G2/G3)	9/46/7	3/29/5		6/17/2
PDC grading (G1/G2/G3)	26/23/13	17/14/6		9/9/7
Tumor budding (present/absent)	32/30	15/22		17/8
LVI (present/absent)	22/40	9/28		13/12

DOID: died of independent diseases; DOD: died of disease; M: male; F: female; LVI: lymphovascular invasion.

**Table 2:** Distribution of KRAS, PIK3CA, and BRAF mutations according to the status of patients.

Mutations	Patients alive/DOID	Patients DOD
KRAS G12C	1	0
KRAS G12D	2	4
KRAS G12D + PIK3CA R108H	0	1
KRAS G12D + G13D + G117N	0	1
KRAS G12F	0	1
KRAS G12R + PIKRCA E545K	0	1
KRAS G12V	2	3
KRAS G13D	5	2
KRAS G13D + PIK3CA R88Q	1	0
KRAS G13D + PIK3CA E545K	0	1
KRAS Q61H	1	0
KRAS A146T	0	1
KRAS A 146T + PIK3CA R108H	0	1
BRAF V600E	1	2
Absent (WT)	24	7

DOID: died of independent disease; DOD: died of disease.

**Table 3:** Statistical correlations between mutational status and the various clinicopathological parameters of the tumors.

	KRAS mutation		*P*	PIK3CA mutation		*P*	*n* mutations/case			*P*
	Absent	Present		Absent	Present		0	1	2	
*Age*										
≤68 years	16	15		28	3		16	11	4	
>68 years	18	13	0.798	29	2	1	15	14	2	0.588
*Gender*										
Male	24	11		35	0		22	20	0	
Female	10	17	**0.02**	22	5	**0.012**	9	12	6	**0.004**
*Size*										
≤3 cms	18	16		31	3		17	14	3	
>3 cms	16	12	0.801	26	2	0.652	14	11	3	0.965
*Site*										
Right colon	9	2		11	0		6	5	0	
Left colon	9	6		15	0		9	5	1	
Rectum	16	20	0.083	31	5	0.14	16	15	5	0.608
*pT*										
T1	1	3		4	0		1	3	0	
T2	33	25	0.319	53	5	1	30	22	6	0.329
*Nodal micrometastases*										
Absent	28	17		44	1		27	17	1	
Present	6	11	0.086	13	4	**0.017**	4	8	5	**0.001**
*Tumor border configuration*										
Expansive	12	6		18	0		11	7	0	
Infiltrative	22	22	0.271	39	5	0.309	20	18	6	0.212
*WHO grade*										
G1	4	5		7	2		4	3	2	
G2	24	22		43	3		24	18	4	
G3	6	1	0.2	7	0	0.202	3	4	0	0.562
*PDC grade*										
G1	23	13		35	1		23	11	2	
G2	7	7		14	0		6	8	0	
G3	4	8	0.168	8	4	0.001	2	6	4	**0.004**
*Tumor budding*										
Absent	17	13		27	3		17	10	3	
Present	17	15	0.803	30	2	0.666	14	15	3	0.541
*LVI*										
Absent	21	12		32	1		20	12	1	
Present	13	16	0.201	25	4	0.176	11	13	5	0.078
*Status*										
Alive, DOID	25	12		36	1		24	12	1	
DOD	9	16	**0.019**	21	4	**0.147**	7	13	5	**0.006**

DOID: died of independent diseases; DOD: died of disease; LVI: lymphovascular invasion.

**Table 4:** Univariate and multivariate analyses for CSS in fifty patients with stage I CRC.

Parameter	Univariate analyses		Multivariate analyses	
	HR (95% CI)	*P*	HR (95% CI)	*P*
*Age*				
≤68 years	1			
>68 years	1.2 (0.5–2.6)	0.642		
*Gender*				
Male	1			
Female	1.2 (0.5–2.8)	0.537		
*Size*				
≤3 cms	1			
>3 cms	0.8 (0.4–1.9)	0.783		
*Site*				
Right colon	1			
Left colon	0.8 (0.2–2.9)			
Rectum	1.9 (0.6–5.5)	0.241		
*pT*				
T1	1			
T2	0.4 (0.07–2.3)	0.139		
*Nodal micrometastases*				
Absent	1			
Present	10.4 (3.6–29.8)	**<0.0001**		
*Tumor border configuration*				
Expansive	1			
Infiltrative	2.6 (1.1–6.1)	0.0542		
*WHO grade*				
G1	1			
G2	0.5 (0.1–1.6)			
G3	0.4 (0.08–2.1)	0.322		
*PDC grade*				
G1	1			
G2	2.6 (1–7.2)			
G3	3.6 (1.1–10.9)	**0.0112**		
*Tumor budding*				
Absent	1			
Present	2.3 (1–5.2)	**0.0334**		
*LVI*				
Absent	1		1	
Present	5.9 (2.6–13.2)	**<0.0001**	6.2 (2.3–16.6)	**0.0003**
*KRAS mutations*				
Absent	1			
Present	2.4 (1.1–5.4)	**0.0228**		
*PIK3CA mutations*				
Absent	1			
Present	3.2 (0.5–17.7)	**0.0214**		
*Number of mutations/case*				
0	1			
1	2.7 (1.1–6.2)			
2/3	5.6 (1.1–27)	**0.039**		

LVI: lymphovascular invasion.

## Data Availability

The data used to support the findings of this study are available from the corresponding author upon request.
